# Enhanced recovery after surgery nursing improves postoperative outcomes in laparoscopic radical nephrectomy: a cumulative meta-analysis

**DOI:** 10.3389/fsurg.2026.1717320

**Published:** 2026-04-10

**Authors:** Yan Wang, Xiaoyan Wang, Yu Gao

**Affiliations:** 1 Nursing Department, Peking University First Hospital Ningxia Women and Children's Hospital (Ningxia Hui Autonomous Region Maternal and Child Health Hospital), Yinchuan, China; 2 School of Nursing, Hexi University, Zhangye, China

**Keywords:** cumulative meta-analysis, enhanced recovery after surgery nursing, eras, nurse, radical nephrectomy

## Abstract

**Background:**

Renal carcinoma is a common malignant tumor of the urinary system worldwide. Given substantial evidence demonstrating the beneficial effects of enhanced recovery after surgery (ERAS) care on recovery following laparoscopic radical nephrectomy for renal cancer, in this study, we conducted a systematic review and meta-analysis to summarize relevant studies and evaluate the application value of ERAS care in this context.

**Methods:**

We searched databases such as PubMed, Embase, The Cochrane Library, Web of Science, OVID, CNKI, Wanfang Data, VIP, and the China Biological Literature Database for clinical studies comparing ERAS care with traditional perioperative care in patients undergoing laparoscopic radical nephrectomy for renal cancer, up to December 2025. Two independent reviewers performed literature screening, data extraction, and quality assessment of the included studies. A cumulative meta-analysis was conducted using Stata version 12.0.

**Results:**

A total of 26 relevant studies were included, comprising 24 randomized controlled studies and two quasi-experimental studies, involving 2,361 patients (1,172 in the ERAS care group and 1,189 in the traditional care group). The cumulative meta-analysis results indicated that patients receiving ERAS care experienced significantly earlier times to first anal exhaust, first feeding, first urination time after surgery, first defecation, catheter encumbrance time, first-time out-of-bed activity, length of hospital stay and removal time of drainage tube postoperatively. Furthermore, and postoperative hospital stay were shorter in the ERAS group. The ERAS group also demonstrated a lower overall incidence of postoperative total complications and higher patient satisfaction.

**Conclusion:**

The application of ERAS care in laparoscopic radical nephrectomy for renal cancer can accelerate postoperative recovery, shorten postoperative hospital stay, and reduce the incidence of postoperative complications. However, because of potential heterogeneity among the included studies, these conclusions warrant further validation by more high-quality research.

**Systematic Review Registration:**

https://www.crd.york.ac.uk/prospero, PROSPERO CRD420251159414.

## 1 Introduction

Renal cell carcinoma (RCC) is one of the most common malignant tumors in the urinary system worldwide, with a continuously rising incidence and mortality rate, posing a significant challenge to public health (1). RCC accounts for approximately 3% of malignant tumors in adults, and its global incidence has been steadily increasing over the past 30 years, with a noticeable upward trend also observed in China (2, 3). The survival rate of RCC is highly dependent on early diagnosis; however, about one-third of patients present with metastasis at the time of initial diagnosis, resulting in a 5-year survival rate of only 12% (4, 5). In recent years, with the development of endourological techniques, laparoscopic radical nephrectomy has become the mainstream surgical approach, demonstrating significant advantages in reducing postoperative complications, enhancing recovery speed, and improving the aesthetic appearance of surgical incisions (6). The combination of laparoscopic radical nephrectomy with enhanced recovery after surgery (ERAS) techniques has become a model for modern surgical treatment, aiming to minimize the trauma and stress response caused by surgery, thereby achieving faster, safer, and more comfortable recovery.

In 1990, Kenlet proposed the concept of ERAS, which aims to implement evidence-based perioperative optimization measures to alleviate the physiological and psychological trauma caused by surgery, with the expectation of achieving rapid recovery (7). ERAS integrates and optimizes multidisciplinary techniques and methods from surgery, anesthesia, nutrition, and rehabilitation, based on evidence from clinical studies, to effectively intervene in perioperative patients, reduce surgical stress, maintain physiological homeostasis, and accelerate patient recovery. This approach is mainly divided into three parts: preoperative, intraoperative, and postoperative interventions (8). Since Kehlet first described the application of ERAS in colorectal surgery in 1997, the ERAS concept has gradually been recognized and adopted by disciplines such as gastrointestinal surgery, cardiovascular surgery, gynecology, and orthopedics (9). It can promote teamwork, enhance patient satisfaction, reduce perioperative complications, shorten hospital stays, and lower hospitalization costs (10, 11). The application of ERAS in urology is relatively recent. A systematic review demonstrated that the ERAS protocol for children undergoing urological reconstruction surgery could reduce the risk of complications and length of hospital stay without increasing the risk of readmission. However, the study population was focused on children, and evidence regarding ERAS in adult urological surgery remains lacking (12). Previous meta-analyses indicated (13, 14) that ERAS care could shorten hospital stay, the time to first defecation, time to first anal exhaust, and time to drainage tube removal, as well as reduce the incidence of nausea in patients undergoing radical prostatectomy. Nevertheless, these two studies lack an analysis of the evolution of evidence. Currently, the application of ERAS concepts in perioperative nursing in urology is still in the process of refinement (15).

In recent years, many scholars have achieved good results in managing and intervening patients undergoing radical nephrectomy for kidney cancer through the ERAS nursing concept to facilitate rapid recovery. Compared with traditional meta-analyses that conduct a single comprehensive analysis at a specific time point, a cumulative meta-analysis allows for the analysis of conditions at various time points. Each included study is sequentially added to a single study based on certain criteria (such as publication time or sample size), enabling multiple meta-analyses, which helps in the early identification of statistically significant interventions. Combined with graphical representations, this meta-analysis can further reflect the dynamic changes in research results and can be used to assess the impact of each study on the overall results (16, 17); thus, this study aims to conduct a cumulative meta-analysis of published clinical trials on the application of ERAS nursing in patients undergoing laparoscopic radical nephrectomy, with the goal of evaluating the impact of ERAS nursing on the recovery outcomes of these patients and providing evidence-based support for clinical nursing practice.

## 2 Methods

This study follows the PRISMA guidelines for systematic reviews and meta-analyses.

### 2.1 Protocol and registration

The evaluation protocol for this study has been prospectively registered in the International Prospective Register of Systematic Reviews (PROSPERO) (CRD420251159414).

### 2.2 Inclusion and exclusion criteria

Inclusion criteria were established as follows:

(a) Participants: Patients undergoing laparoscopic radical nephrectomy, regardless of disease type.
(b) Interventions: The experimental group received ERAS nursing.
(c) Comparisons: The control group received traditional rehabilitation surgical care.
(d) Outcomes: The primary outcome measure is total complications (refer to the totality of any newly occurring adverse events related to laparoscopic radical nephrectomy or perioperative care, which occur during hospitalization and within 30 days after discharge); the secondary outcome measures include the time to first flatus, time to first feeding, time to spontaneous urination, time to drain removal, length of hospital stay, duration of urethral catheterization, satisfaction, and time to first bowel movement. Satisfaction was assessed using an investigator-developed nursing satisfaction questionnaire, with responses categorized on a three-point Likert scale: “very satisfied,” “satisfied,” and “dissatisfied.” The overall nursing satisfaction rate was calculated using the following formula: (number of “very satisfied” + number of “satisfied”)/total number of cases × 100%.
Study design: randomized controlled trials (RCTs) and quasi-experimental studies, including both prospective and retrospective studies.
The quality assessment of included literature is A or B, with no restriction on the language of the literature.

Exclusion criteria comprised the following:

(a) Articles without full text or data that cannot be extracted.
(b) Duplicate literature.
(c) Conference abstracts.
(d) The experimental group received ERAS nursing combined with other nursing methods.

### 2.3 Search strategy

Databases such as PubMed, Embase, The Cochrane Library, Web of Science, OVID, CNKI, Wanfang Data, VIP, and China Biological Literature Database were searched comprehensively for studies comparing ERAS rehabilitation nursing with traditional rehabilitation nursing from the time of establishment of the database to 1 December 2025. The search strategy combined subject terms with free words, supplemented by manual searches for relevant meta-analyses or systematic reviews. Search terms included “ERAS,” “enhanced postsurgical recovery,” “recovery, enhanced postsurgical,” “fast track surgery,” “ERAS,” “FTS,” “Carcinoma, Renal Cell,” “RCCs,” “Renal Carcinoma*,” “Nephroid Carcinoma*,” etc. There were no language restrictions. Taking the specific search strategy in Web of Science as an example, the following can be obtained:

#1 TS = Enhanced Recovery After Surgery OR enhanced postsurgical recovery OR recovery, enhanced postsurgical OR fast track surgery OR ERAS OR FTS #2 TS = Carcinoma, Renal Cell OR Renal Cell Carcinomas OR Renal Carcinoma* OR Nephroid Carcinoma* OR Renal Cell Adenocarcinoma* OR Renal Cell Carcinoma OR Renal Cell Cancer* OR Renal Adenocarcinoma* OR Adenocarcinoma Of Kidney* #3 #1 AND #2.

### 2.4 Literature selection

All downloaded references were imported into EndNote ×8 reference management software to exclude duplicate literature. Two researchers (YW and XW) independently screened the literature based on inclusion criteria by reading titles, abstracts, and full texts sequentially. For literature that met the inclusion criteria, the full text was read for judgment. In case of disagreement, a third party was invited to discuss and resolve the issue.

### 2.5 Data extraction

Data extraction from the included studies was conducted by two researchers (YW and XW), followed by cross-checking. In case of discrepancies, a third researcher conducted the analysis and made the final decision. A predesigned form was used to collect data from the included literature, extracting information such as basic literature information (title, authors, and publication year); basic characteristics of the study subjects (year, sample size, inclusion sample time range, design, interventions, etc.); key elements for bias risk assessment; and data for all outcome indicators.

### 2.6 Quality assessment

Two researchers (YW and XW) independently assessed the quality of the selected studies and cross-validated the results. The bias assessment tool for RCTs was based on the Cochrane Handbook version 5.1.0 (18). The quality assessment tool for quasi-experimental studies used the JBI evidence–based health center's quality assessment tool (19).

### 2.7 Statistical analysis

Stata 12.0 statistical software was used for performing a conventional meta-analysis and the cumulative meta-analysis. Continuous data were analyzed using WMD as the effect measure, while count data used RR as the effect measure, with each effect size providing its 95% CI. The heterogeneity among the included studies was assessed using *I*^2^ to determine the degree of heterogeneity. Meta-analyses were performed using a random-effects model accounting for clinical heterogeneity (20). When significant heterogeneity was present among studies, a subgroup analysis was conducted to explore the sources of heterogeneity. The significance level for the meta-analysis was set at *α* = 0.05. For cumulative meta-analysis, heterogeneity tests were first conducted using *Q* and *I*² statistics on the combined studies; then, the studies were sorted by publication date, and the corresponding effect models were applied for cumulative meta-analysis to evaluate whether the differences in outcome effects were statistically significant. To further examine the temporal variability of the “combined effect” (stability of effect results), a generalized least squares (GLS) regression strategy was employed for the time trend test of cumulative meta-analysis, ordered by publication date. When the *P*-values from both tests were >0.05, it was considered that the trend did not exhibit temporal variability, indicating stable combined effect results.

To evaluate the stability of the results, sensitivity analyses were conducted for all outcome indicators. By sequentially excluding studies and performing meta-analysis on the remaining studies, changes in the combined results were observed to determine whether the results were significantly influenced by certain studies. Publication bias was assessed using the Egger test and Begg's test.

GRADE is used to assess the overall quality of the evidence and the strength of the recommendations (21). This standard classifies the quality of evidence into four categories: very low, low, medium, and high.

## 3 Results

### 3.1 Literature screening process and results

**Figure 1:**
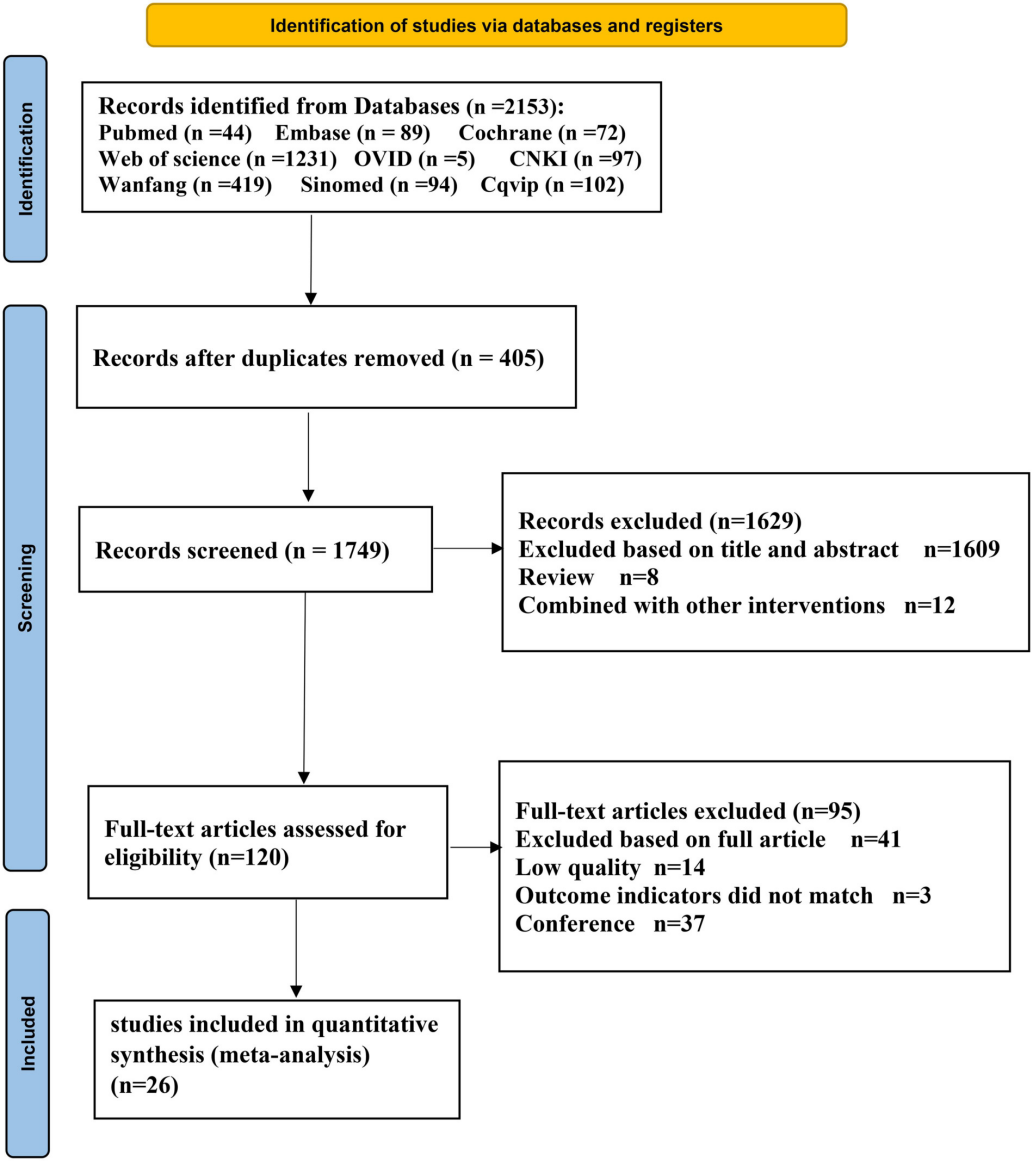
PRISMA flow diagram.

A total of 2,155 articles were initially obtained, and after removing duplicates, 1,750 articles were further screened based on their titles and abstracts. A total of 1,609 articles deemed irrelevant to the research topic were excluded, along with eight review articles and 12 articles discussing laparoscopic surgery combined with other interventions, resulting in 121 articles meeting the criteria for full-text evaluation. A total 41 articles were excluded after full-text screening, three due to non-compliance with outcome indicators, 37 were conference papers, and 14 were identified as low quality, leaving 26 studies included in the research (22–47). The process and results of literature selection are illustrated in Figure 1.

### 3.2 Basic characteristics of the included studies

Table 1 describes the main characteristics of the 26 studies included in this analysis, covering publications from 2015 to 2025. Two studies were quasi-experimental studies, while 24 studies were randomized controlled studies. All included studies were from China. The sample size of the intervention group was 1,172, while the control group had 1,189 participants. The intervention employed ERAS nursing, while the control group used conventional rehabilitation surgical nursing.

**Table 1:** Characteristics of the included trials.

Author	Sample (T/C)	Year (T/C)	Design	Included in the sample time range	Intervention measure	Control measure	Type of surgery	Outcome
Wang et al. (22)	60/60	58.3 ± 6.7/57.9 ± 7.2	RCT	2023–2024	ERAS	Traditional nursing	LRN	①②⑥⑩
Wang et al. (23)	120/120	65.8 ± 7.1/64.6 ± 6.5	RCT	September 2020–September 2022	ERAS	Traditional nursing	LRN	①②④⑥⑩
Shi et al. (24)	50/50	46.29 ± 3.77/45.64 ± 3.91	RCT	January 2017–January 2020	ERAS	Traditional nursing	LRN	①②③④⑧⑩
Shi et al. (25)	59/59	41.28 ± 4.13/42.45 ± 4.39	RCT	January 2019–January 2021	ERAS	Traditional nursing	LRN	①②⑤⑥⑦⑩
Huang (26)	44/44	50.12 + 0.65/53.47 + 0.53	RCT	June 2019–July 2021	ERAS	Traditional nursing	LRN	①②③⑤⑧⑩
Sun et al. (27)	35/33	58 ± 11/58 ± 12	q-RCT	January 2017–October 2020	ERAS	Traditional nursing	RLRN	①②③⑤⑥⑦⑧⑩
Li et al. (29)	62/58	57.28 ± 15.6/56.85 ± 14.4	RCT	October 2018–October 2020	ERAS	Traditional nursing	LRN	①②③⑤⑥⑦⑧⑩
Li et al. (28)	50/50	/	RCT	June 2015–June 2020	ERAS	Traditional nursing	LRN	①②⑥⑧⑨
Li et al. (30)	30/30	/	RCT	/	ERAS	Traditional nursing	LRN	②⑥⑩
Wang et al. (31)	30/30	63.45 ± 5.23/62.45 ± 5.35	RCT	January 2018–October 2019	ERAS	Traditional nursing	LRN	①②③④⑥⑩
Liu et al. (32)	58/58	58.6 ± 4.3/54.5 ± 8.8	RCT	January 2015–January 2019	ERAS	Traditional nursing	LRN	②③⑥⑦⑧⑩
Liu et al. (33)	51/51	54.2 ± 12.7/55.4 ± 10.8	q-RCT	January–December 2019	ERAS	Traditional nursing	RLRN	①
Huang and Zhang (34)	25/25	54.6 ± 8.6/54.8 ± 7.2	RCT	July 2015–December 2018	ERAS	Traditional nursing	RLRN	②③⑤⑥⑩
Chen et al. (35)	46/46	64.2 ± 6.5/63.8 ± 7.8	RCT	October 2017–March 2019	ERAS	Traditional nursing	LRN	①②③⑥⑧⑩
Liu and Wang (36)	30/30	55.70 ± 9.32/54.95 ± 10.08	RCT	February 2017–January 2018	ERAS	Traditional nursing	LRN	②③⑤⑥⑩
Gong et al. (37)	25/22	55.0512.55/52.5 ± 10.49	RCT	January 2016–September 2017	ERAS	Traditional nursing	LRN	②③⑤⑥⑨
Zhang et al. (38)	62/88	65.23 ± 6.15/67.12 ± 5.15	RCT	January 2013–December 2016	ERAS	Traditional nursing	LRN	①②⑤⑥⑧
Yao et al. (39)	57/57	53.42 ± 13.24/52.3 ± 12.48	RCT	July 2016–September 2017	ERAS	Traditional nursing	RLRN	①②③⑥⑩
Wang et al. (40)	39/39	49.35 ± 3.60/49.27 ± 3.68	RCT	December 2015–December 2017	ERAS	Traditional nursing	LRN	①②③⑥⑧⑩
Liu et al. (41)	19/19	65.06 ± 9.14	RCT	February 2015–August 2016	ERAS	Traditional nursing	LRN	②⑤⑥
Gong et al. (42)	28/27	/	RCT	June 2014–October 2017	ERAS	Traditional nursing	RLRN	②③⑤⑥⑦
Zheng et al. (43)	31/32	56.45 ± 3.52/55.32 ± 4.3	RCT	June–December 2016	ERAS	Traditional nursing	LRN	①③⑤⑥⑦⑧⑩
Wang et al. (44)	48/48	47.1 ± 4.3/46.7 ± 4.6	RCT	January 2015–June 2016	ERAS	Traditional nursing	LRN	①②⑥⑧⑨⑩
Ma et al. (45)	43/43	75.2 ± 9.3/76.8 ± 8.2	RCT	December 2014–December 2015	ERAS	Traditional nursing	LRN	①②⑤⑥⑦⑨⑩
Chai et al. (46)	35/35	45/42	RCT	January–October 2016	ERAS	Traditional nursing	LRN	②⑤⑥⑦⑨⑩
Xue et al. (47)	35/35	/	RCT	January 2012–December 2014	ERAS	Traditional nursing	RLRN	①②③⑤⑥⑦⑩

① Total complications; ② time of first anal exhaust; ③ time to first feeding; ④ first urination time after surgery; ⑤ removal time of drainage tube; ⑥ length of hospital stay; ⑦ catheter encumbrance time; ⑧ satisfaction; ⑨ time to first defecation; ⑩ first-time out-of-bed activity T, ERAS; C, traditional nursing; LRN, laparoscopic radical nephrectomy; RLRN, retroperitoneal laparoscopic radical nephrectomy; q-RCT, quasi-experimental studies; RCT, randomized controlled studies.

### 3.3 Assessment of bias risk in included studies

The risk of bias in the included studies shows (see Tables 2, 3) that 12 RCTs only mentioned “randomization” without specifying the randomization method, while 11 studies used random number tables or drawing lots. Only one study used reported allocation concealment and single blinding. In quasi-experimental studies, two studies lacked multidimensional outcome measurements before and after the intervention.

**Table 2:** Methodological quality evaluation of 24 included randomized controlled trials.

Study ID	Random sequence generation	Allocation concealment	Blinding of participants and personnel	Blinding of outcome assessment	Incomplete outcome data	Selective reporting	Other bias
Wang et al. (22)	Low	Unclear	High	Unclear	Low	Low	Low
Wang et al. (23)	Low	Low	High	Unclear	Low	Low	Low
Shi et al. (24)	High	Unclear	High	Unclear	Low	Low	Low
Shi et al. (25)	Low	Unclear	High	Unclear	Low	Low	Low
Huang (26)	High	Unclear	Low	Unclear	Low	Low	Low
Li et al. (29)	High	Unclear	High	Unclear	Low	Low	Low
Li et al. (28)	Low	Unclear	High	Unclear	Low	Low	Low
Li et al. (30)	High	Unclear	High	Unclear	Low	Low	Low
Wang et al. (31)	Low	Unclear	High	Unclear	Low	Low	Low
Liu et al. (32)	High	Unclear	High	Unclear	Low	Low	Low
Huang and Zhang (34)	Low	Unclear	High	Unclear	Low	Low	Low
Chen et al. (35)	Low	Unclear	High	Unclear	Low	Low	Low
Liu and Wang (36)	Low	Unclear	High	Unclear	Low	Low	Low
Gong et al. (37)	High	Unclear	High	Unclear	Low	Low	Low
Zhang et al. (38)	High	Unclear	High	Unclear	Low	Low	Low
Yao et al. (39)	Low	Unclear	High	Unclear	Low	Low	Low
Wang et al. (40)	Low	Unclear	High	Unclear	Low	Low	Low
Liu et al. (41)	High	Unclear	High	Unclear	Low	Low	Low
Gong et al. (42)	High	Unclear	High	Unclear	Low	Low	Low
Zheng et al. (43)	High	Unclear	High	Unclear	Low	Low	Low
Wang et al. (44)	High	Unclear	High	Unclear	Low	Low	Low
Ma et al. (45)	Low	Unclear	High	Unclear	Low	Low	Low
Chai et al. (46)	High	Unclear	High	Unclear	Low	Low	Low
Xue et al. (47)	High	Unclear	High	Unclear	Low	Low	Low

**Table 3:** Methodological quality evaluation of two included quasi-experimental studies.

Study ID	Item 1	Item 2	Item 3	Item 4	Item 5	Item 6	Item 7	Item 8	Item 9
Sun et al. (27)	Yes	Yes	Yes	Yes	No	Yes	Yes	Yes	Yes
Liu et al. (33)	Yes	Yes	Yes	Yes	No	Yes	Yes	Yes	Yes

① Is it clear in the study what is the cause and what is the effect?

② Were the participants included in any comparisons similar?

③ Were the participants included in any comparisons receiving similar treatment/care, other than the exposure or intervention of interest?

④ Was there a control group?

⑤ Were there multiple measurements of the outcome both before and after the intervention/exposure?

⑥ Was follow-up complete and if not, were differences between groups in terms of their follow-up adequately described and analyzed?

⑦ Were the outcomes of participants included in any comparisons measured the same way?

⑧ Were outcomes measured in a reliable way?

⑨ Was appropriate statistical analysis used?

### 3.4 Meta-analysis outcomes

#### 3.4.1 Total complications

**Figure 2:**
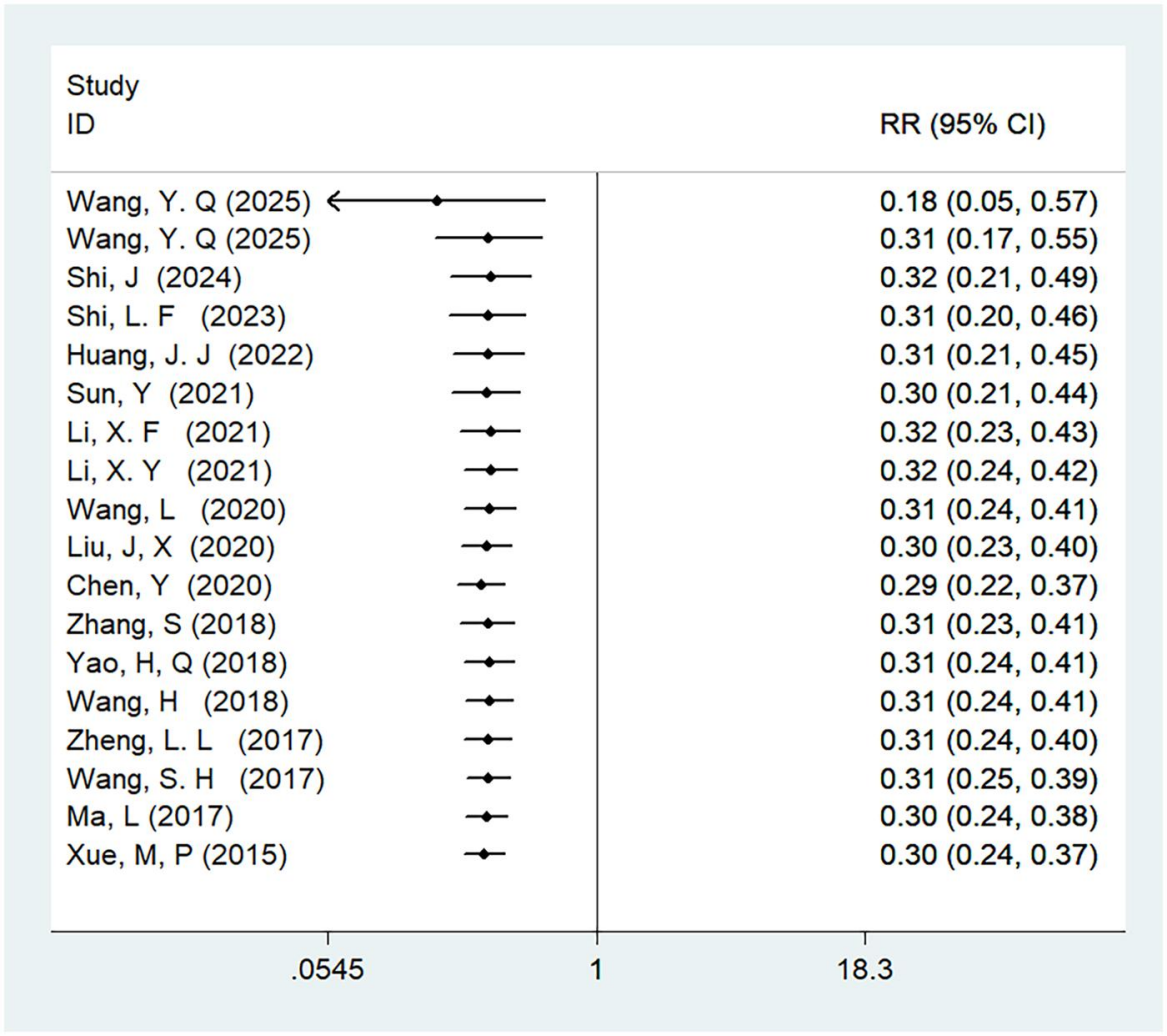
Cumulative meta-analysis forest map of total complications.

**Figure 3:**
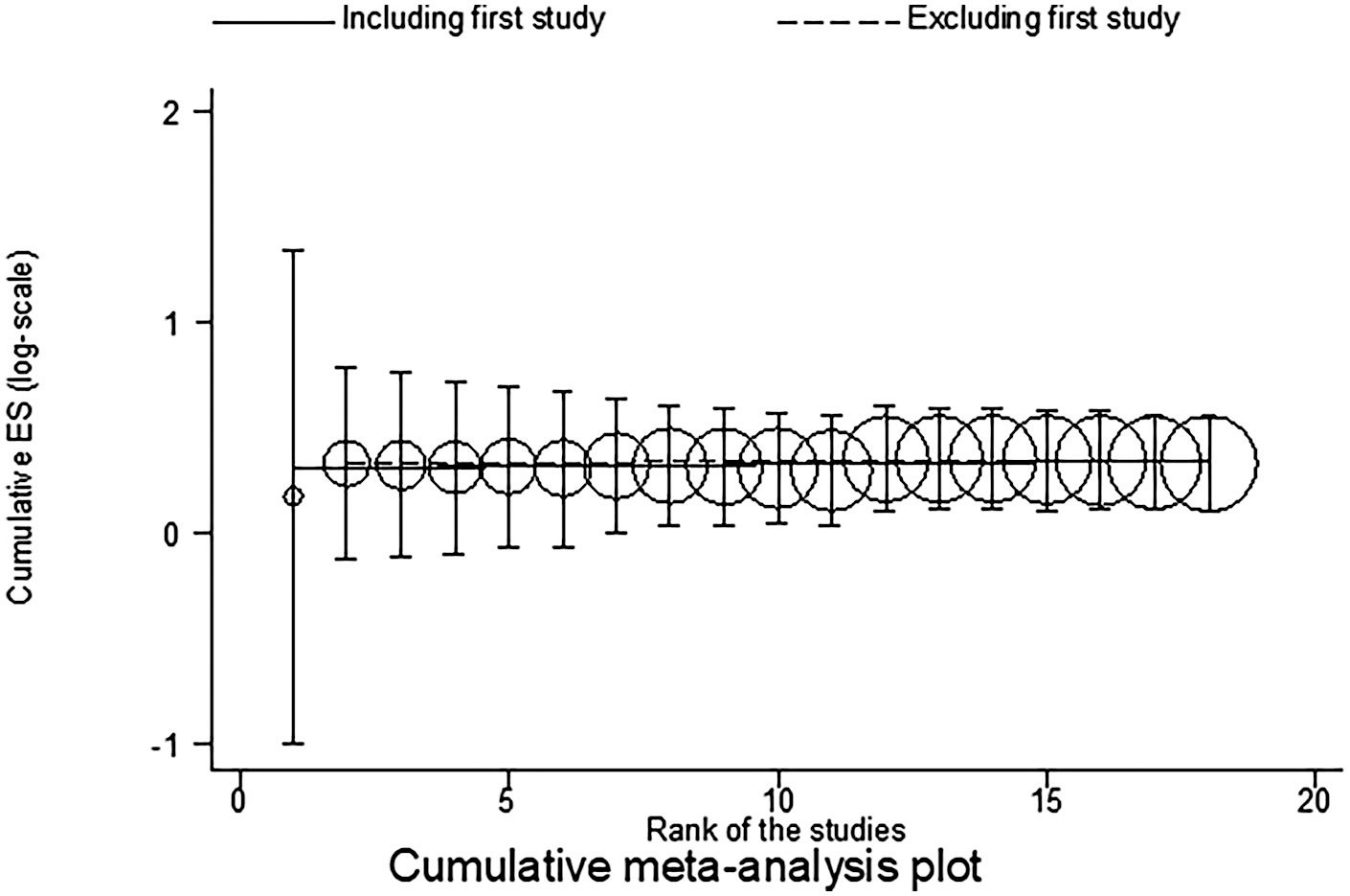
Trend analysis for the cumulative meta-analysis of total complications.

A total of 18 studies were included to analyze total complications, involving 1,865 patients. The results showed no heterogeneity (*I*^2^ = 0%, *P* = 0.45), and there was a statistically significant difference in total complications between ERAS care and conventional care (RR = 0.30, 95% CI = 0.24, 0.37; *P* < 0.05, see Table 4). After cumulative analysis in chronological order of publication, both the RR value and the 95% CI tended to stabilize and showed a good trend of change. The first time when a statistically significant difference in the incidence of total complications was confirmed was in 2015 (RR = 0.30, CI = 0.24, 0.37), and subsequent studies narrowed the confidence interval, gradually increasing the precision of the effect size. This indicates that ERAS care is superior to conventional care in reducing total complications. GLS regression tests showed that including all studies resulted in *P* = 0.05, while removing the first study resulted in *P* = 0.55. This suggests the presence of temporal variability in the results, indicating that the findings are not yet stable and necessitate further validation through additional clinical trials, as detailed in Table 6 and Figures 2, 3.

**Table 4:** Meta-analysis of ERAS VS traditional nursing.

Outcome	Number of studies included	Number of cases	RR/SMD (95% CI）	*P*	Heterogeneity	
					*I* ^2^	*P*
Total complications	18	1865	0.30 (0.24, 0.37)	<0.001	0	0.45
Time of first anal exhaust	24	2,232	−1.89(−2.27, −1.51)	<0.001	93	<0.001
Time to first feeding	15	1,181	−3.67 (−4.64, −2.71)	<0.001	97	<0.001
First urination time after surgery	3	400	−1.70(−2.04, −1.35)	<0.001	43	0.17
Removal time of drainage tube	14	1,119	−3.33(−4.24, −2.43)	<0.001	97	<0.001
Length of hospital stay	23	2,071	−2.09(−2.63, −1.56)	<0.001	96	<0.001
Catheter encumbrance time	9	766	−2.85(−3.84, −1.86)	<0.001	96	<0.001
Satisfaction	10	1,008	1.16 (1.11, 1.21)	<0.001	0	0.88
Time to first defecation	5	399	−1.80(−2.45, −1.16)	<0.001	86	<0.001
First-time out-of-bed activity	20	1,869	−3.69(−4.47, −2.91)	<0.001	97	<0.001

##### 3.4.1.1 Subgroup analysis

Subgroup analyses stratified by study year and surgical type were conducted. The results indicated that ERAS care maintained a significant effect across different years and types of surgery, as detailed in Table 5.

**Table 5:** Subgroup analysis of complications.

Outcome	Number	Heterogeneity test		Effect model	Meta-analysis results	
		*I*^2^ (%)	*P*		RR(95% CI)	*P*
Study year						
2015–2020	10	38	0.11	Random	0.25 (0.16,0.40)	<0.01
2021–2025	8	0	0.96	Random	0.32 (0.24,0.42)	<0.01
Type of surgery						
Laparoscopic radical nephrectomy	14	15	0.28	Random	0.30 (0.23,0.40)	<0.01
Retroperitoneal laparoscopic radical nephrectomy	4	0	0.82	Random	0.22 (0.13,0.39)	<0.01

#### 3.4.2 Time to first anal exhaust

**Figure 4:**
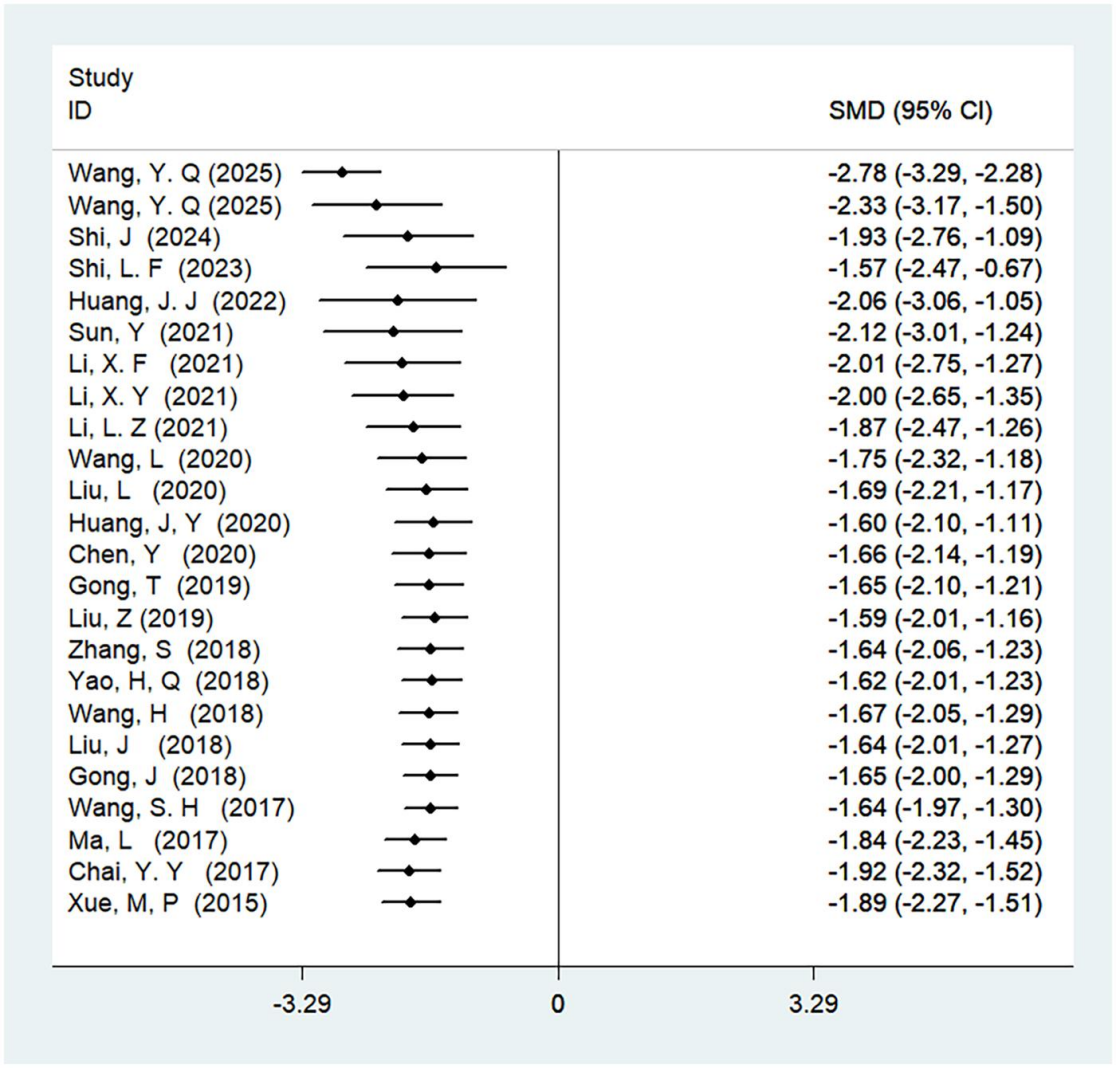
Cumulative meta-analysis forest map of the time to first anal exhaust.

A total of 24 studies were included to analyze the time to first anal exhaust, involving 2,232 patients. The results showed high heterogeneity (*I*^2^ = 93%, *P* < 0.01), and there was a statistically significant difference in the time to first anal exhaust between ERAS care and conventional care (SMD = −1.89, 95% CI = −2.27, −1.51; *P* < 0.05, see Table 4). After cumulative analysis in chronological order of publication, both the SMD value and the 95% CI tended to stabilize and showed a good trend of change. The first time when a statistically significant difference in shortening the time to first anal exhaust was confirmed was in 2015 [SMD = −1.89 (−2.27, −1.51)], and subsequent studies narrowed the confidence interval, gradually increasing the precision of the effect size. This indicates that ERAS care is superior to conventional care in shortening the time to first anal exhaust. GLS regression tests indicated that including all studies resulted in *P* = 0.00, while removing the first study resulted in *P* = 0.07. This suggests the presence of temporal variability in the results, indicating that the findings are not yet stable and necessitate further validation through additional clinical trials, as detailed in Table 6, Figure 4, and Supplementary Material S1.

**Table 6:** Trend of cumulative meta-analysis.

Outcome	Publication time (year)	Effect size (RR/WMD)	GLS regression test (*P*)
Total complications	2015–2025	0.30 (0.24, 0.37)	0.05*/0.55**
Time of first anal exhaust	2015–2025	−1.89(−2.27, −1.51)	0.00*/0.07**
Time to first feeding	2015–2024	−3.67(−4.64, −2.71)	0.00*/0.48**
First urination time after surgery	2015–2023	−3.33(−4.24, −2.43)	0.24*/0.00**
Length of hospital stay	2015–2025	−2.09(−2.63, −1.56)	0.00*/0.00**
Catheter encumbrance time	2015–2023	−2.85(−3.84, −1.86)	0.01*/0.02**
Satisfaction	2017–2024	1.16 (1.11, 1.21)	0.00*/0.20**
Time to first defecation	2017–2021	−1.80(−2.45, −1.16)	0.00*/0.00**
First-time out-of-bed activity	2015–2025	−3.69(−4.47, −2.91)	0.00*/0.00**

* Indicates the *P*-value that includes all studies.

** indicates the removal of the *P*-value of the first study.

#### 3.4.3 Time to first feeding

**Figure 5:**
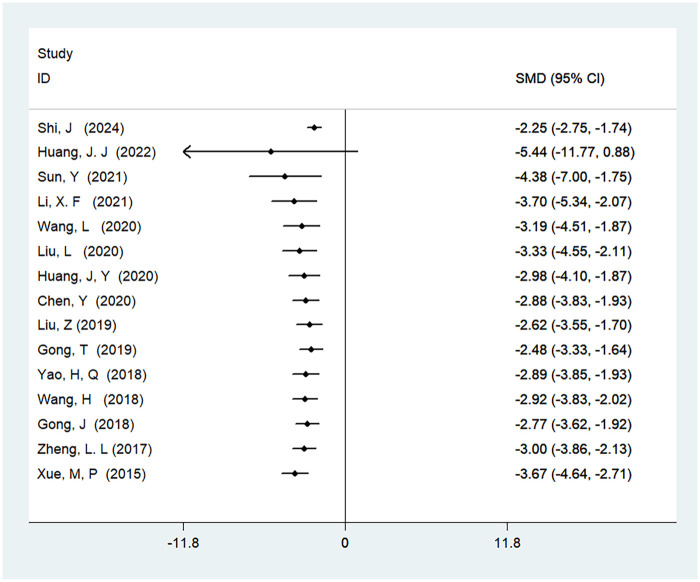
Cumulative meta-analysis forest map of the time to first feeding.

A total of 15 studies were included to analyze the time to first feeding, involving 1,181 patients. The results demonstrated high heterogeneity (*I*^2^ = 97%, *P* < 0.01), and there was a statistically significant difference in the time to first feeding between ERAS care and conventional care (SMD = −3.67, 95% CI = −4.64, −2.71; *P* < 0.05, see Table 4). After cumulative analysis in chronological order of publication, both the SMD value and the 95% CI tended to stabilize and showed a good trend of change. The first time when a statistically significant difference in shortening the time to first feeding was confirmed was in 2015 [SMD = −3.67 (−4.66, −2.71)], and subsequent studies narrowed the confidence interval, gradually increasing the precision of the effect size. This indicates that ERAS care is superior to conventional care in shortening the time to first feeding. GLS regression tests showed that including all studies resulted in *P* = 0.00, while removing the first study resulted in *P* = 0.48. This suggests the presence of temporal variability in the results, indicating that the findings are not yet stable and necessitate further validation through additional clinical trials, as detailed in Table 6, Figure 5, and Supplementary Material S2.

#### 3.4.4 First urination time after surgery

**Figure 6:**
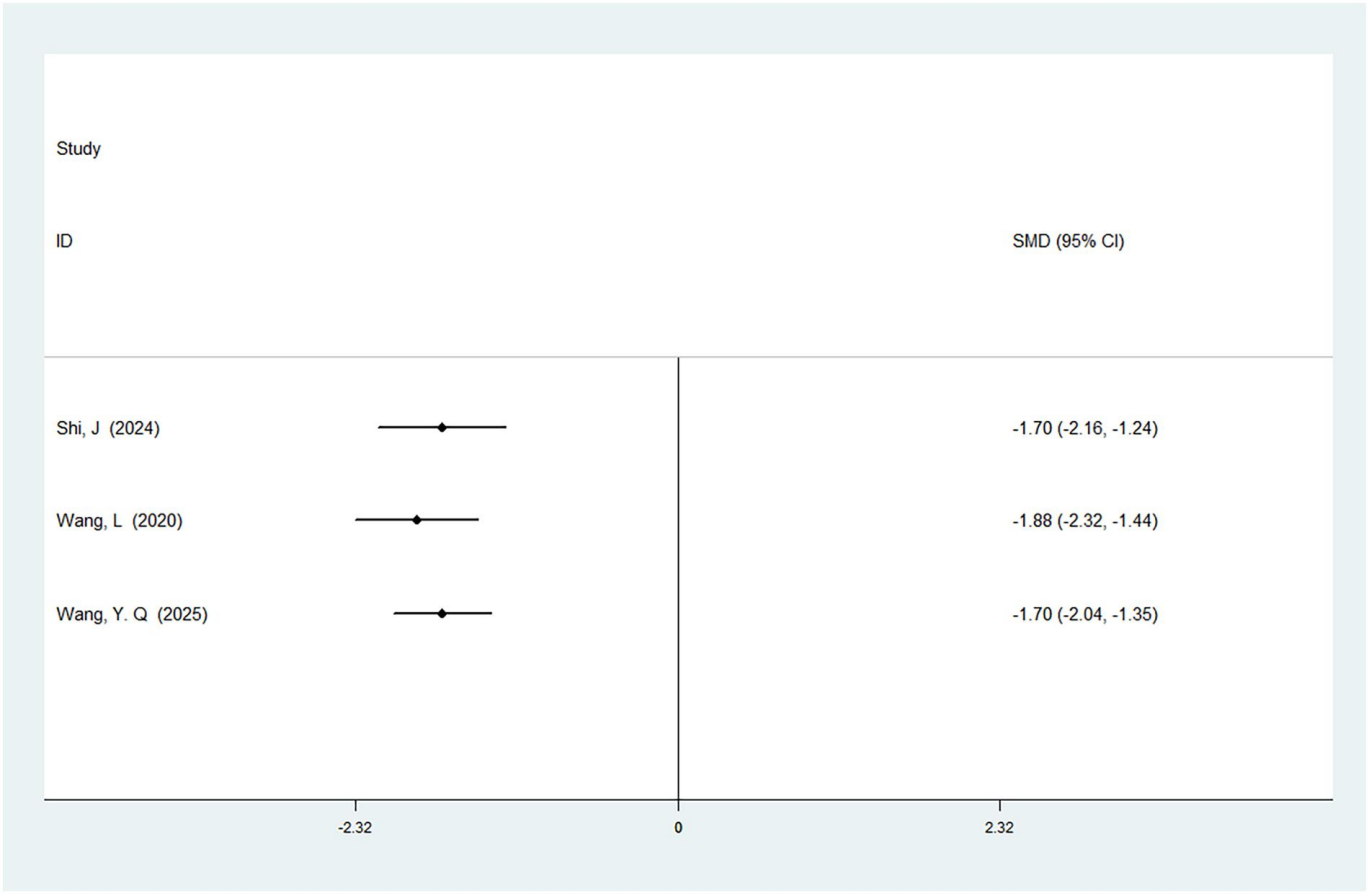
Cumulative meta-analysis forest map of urination time after surgery.

A total of three studies were included in the analysis of first urination time after surgery, involving 400 patients. The results displayed no heterogeneity (*I*^2^ = 43%, *P* = 0.17), and there was a statistically significant difference in the first urination time after surgery between ERAS care and conventional care (SMD = −1.70, 95% CI = −2.04, −1.35; *P* < 0.05, see Table 4). After cumulative analysis in chronological order of publication, the SMD value and the 95% CI tended to stabilize and showed a good trend of change. The first time when a statistically significant difference in shortening the first urination time after surgery was confirmed was in 2015 [SMD = −1.70 (−2.04,−1.35)]. Subsequent studies narrowed the confidence interval, and the precision of the effect size gradually increased. This indicates that ERAS care is superior to conventional care in shortening the first urination time after surgery. The GLS regression test indicated that including all studies resulted in *P* = 0.24. This suggests the presence of temporal variability in the results, indicating that the findings are not yet stable and necessitate further validation through additional clinical trials, as detailed in Table 6, Figure 6, and Supplementary Material S3.

**Figure 7:**
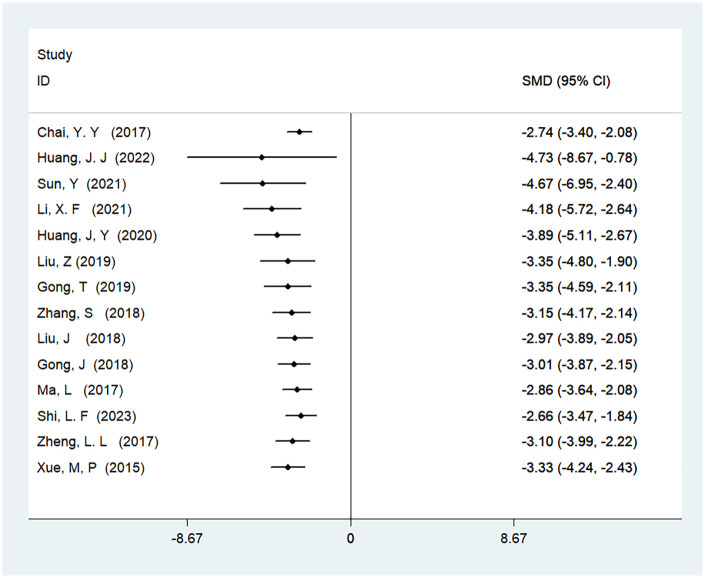
Cumulative meta-analysis forest map of removal time of drainage tube.

#### 3.4.5 Removal time of drainage tube

A total of 14 studies were included in the analysis of the removal time of drainage tube, involving 1,119 patients. The results depicted high heterogeneity (*I*^2^ = 97%, *P* < 0.01), and there was a statistically significant difference in the removal time of drainage tube between ERAS care and conventional care (SMD=−3.33, 95% CI = −4.24, −2.43; *P* < 0.05, see Table 4). After cumulative analysis in chronological order of publication, the SMD value and the 95% CI tended to stabilize and demonstrated a good trend of change. The first time when a statistically significant difference in shortening removal time of drainage tube was confirmed was in 2015 [SMD = −3.33(−4.24,−2.43)]. Subsequent studies narrowed the confidence interval, and the precision of the effect size gradually increased. Because of the limited data, GIS regression tests could not be conducted. This shows that ERAS care is superior to conventional care in the removal time of drainage tube, as detailed in Figure 6 and Supplementary Material S4.

#### 3.4.6 Length of hospital stay

**Figure 8:**
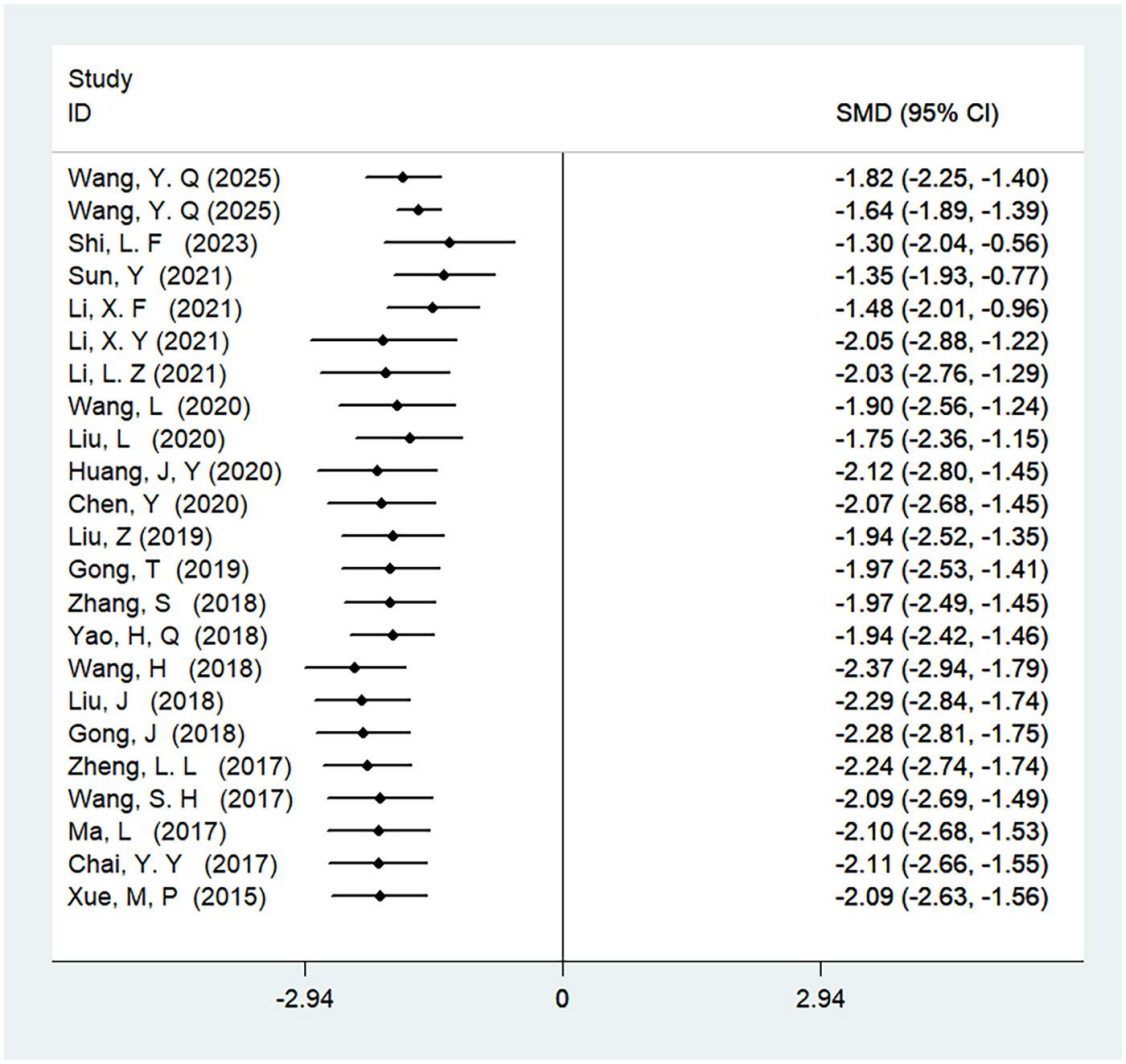
Cumulative meta-analysis forest map of length of hospital stay.

A total of 23 studies were included in the analysis of the length of hospital stay, involving 2,071 patients. The results showed high heterogeneity (*I*^2^ = 96%, *P* < 0.01), and there was a statistically significant difference in the length of hospital stay between ERAS care and conventional care (SMD = −2.09, 95% CI = −2.63, −1.56; *P* < 0.05, see Table 4). After cumulative analysis in chronological order of publication, the SMD value and the 95% CI tended to stabilize and showed a good trend of change. The first time when a statistically significant difference in shortening the length of hospital stay was confirmed was in 2015 [SMD = −2.09 (−2.63, −1.56)]. Subsequent studies narrowed the confidence interval, and the precision of the effect size gradually increased. This demonstrates that ERAS care is superior to conventional care in the length of hospital stay. The GLS regression test indicated that including all studies resulted in *P* = 0.00, and removing the first study resulted in *P* = 0.00. This suggests the presence of temporal variability in the results, indicating that the findings are not yet stable and necessitate further validation through additional clinical trials, as detailed in Table 6, Figure 8, and Supplementary Material S5.

#### 3.4.7 Catheter encumbrance time

**Figure 9:**
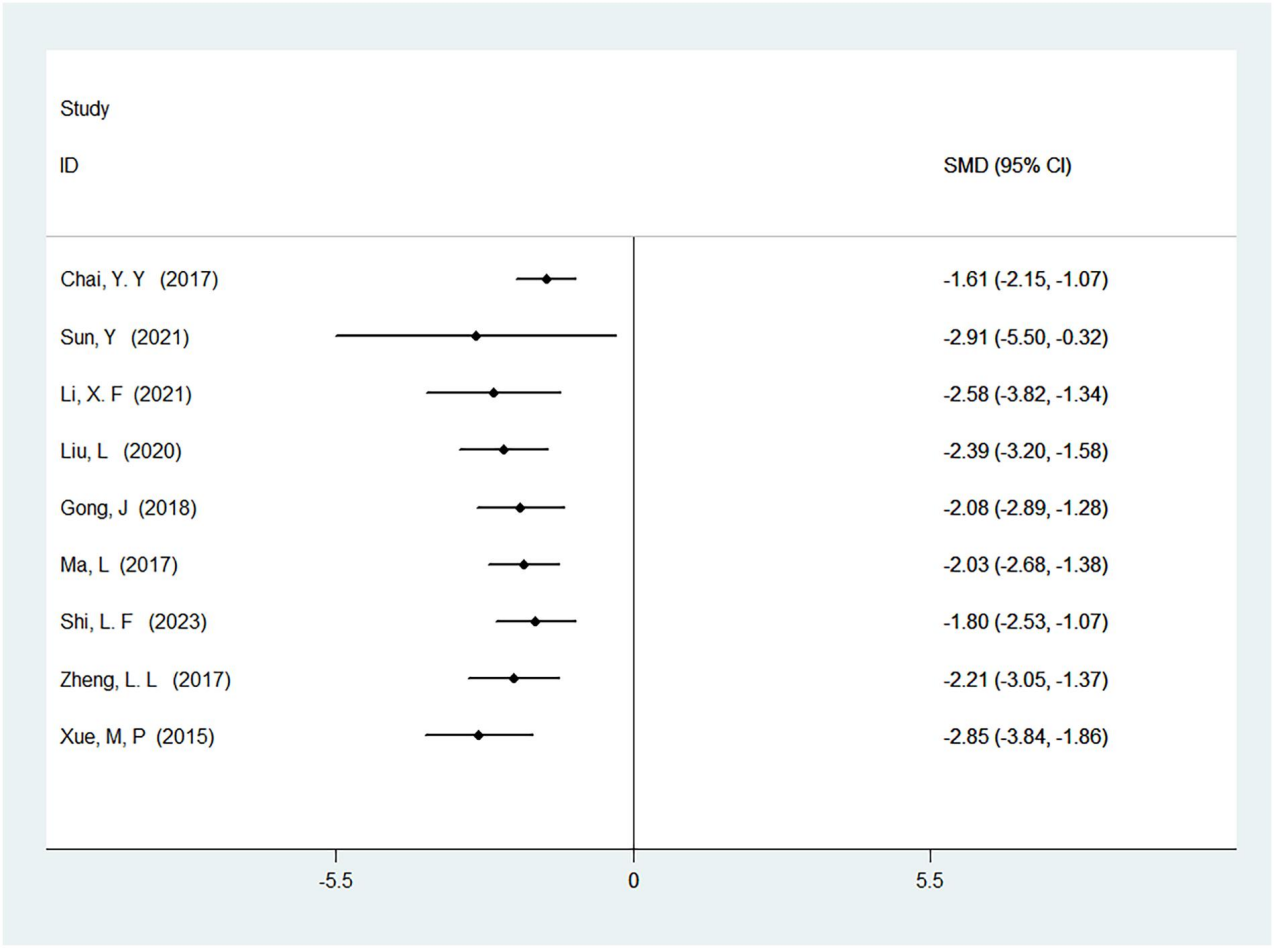
Cumulative meta-analysis forest map of catheter encumbrance time.

A total of nine studies were included in the analysis of the catheter encumbrance time, involving 766 patients. The results indicated high heterogeneity (*I*^2^ = 96%, *P* < 0.01), and there was a statistically significant difference in the catheter encumbrance time between ERAS care and conventional care (SMD = −2.85, 95% CI = −3.84, −1,86; *P* < 0.05, see Table 4). After cumulative analysis in chronological order of publication, the SMD value and the 95% CI tended to stabilize and showed a good trend of change. The first time when a statistically significant difference in shortening catheter encumbrance time was confirmed was in 2015 [SMD = −2.85 (−3.84, −1,86)]. Subsequent studies narrowed the confidence interval, and the precision of the effect size gradually increased. This shows that ERAS care is superior to conventional care in shortening the catheter encumbrance time. The GLS regression test showed that including all studies resulted in *P* = 0.01, and removing the first study resulted in *P* = 0.02. This suggests the presence of temporal variability in the results, indicating that the findings are not yet stable and necessitate further validation through additional clinical trials, as detailed in Table 6, Figure 9, and Supplementary Material S6.

#### 3.4.8 Satisfaction

**Figure 10:**
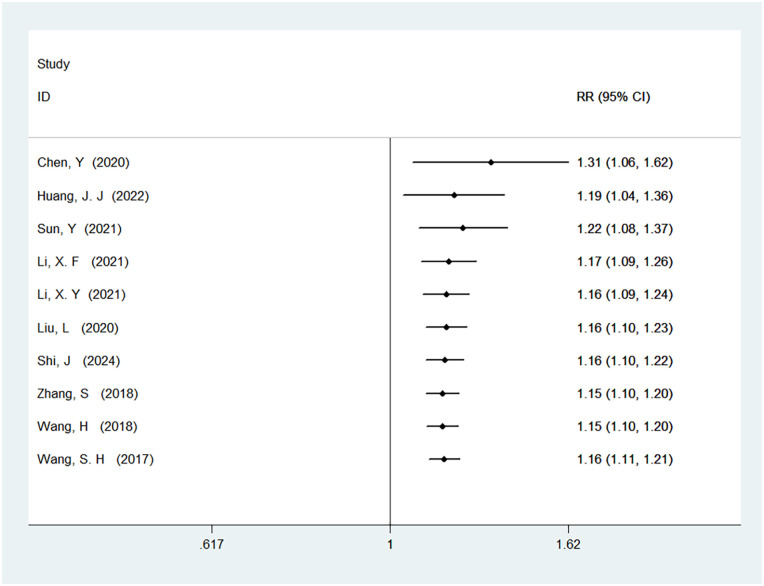
Cumulative meta-analysis forest map of satisfaction.

A total of 10 studies were included in the analysis of satisfaction, involving 1,008 patients. The results showed no heterogeneity (*I*^2^ = 0%, *P* = 0.88), and there was a statistically significant difference in satisfaction between ERAS care and conventional care (RR = 1.16, 95% CI = 1.11, 1.21; *P* < 0.05, see Table 4). After cumulative analysis in chronological order of publication, the RR value and the 95% CI tended to stabilize and showed a good trend of change. The first time when a statistically significant difference in improving satisfaction was first confirmed was in 2017 [RR = 1.16, CI = 1.11, 1.21]. Subsequent studies narrowed the confidence interval, and the precision of the effect size gradually increased. This demonstrates that ERAS care is superior to conventional care in improving satisfaction. The GLS regression test indicated that including all studies resulted in *P* = 0.00, and removing the first study resulted in *P* = 0.20. This suggests the presence of temporal variability in the results, indicating that the findings are not yet stable and necessitate further validation through additional clinical trials, as detailed in Table 6, Figure 10, and Supplementary Material S7.

#### 3.4.9 Time to first defecation

**Figure 11:**
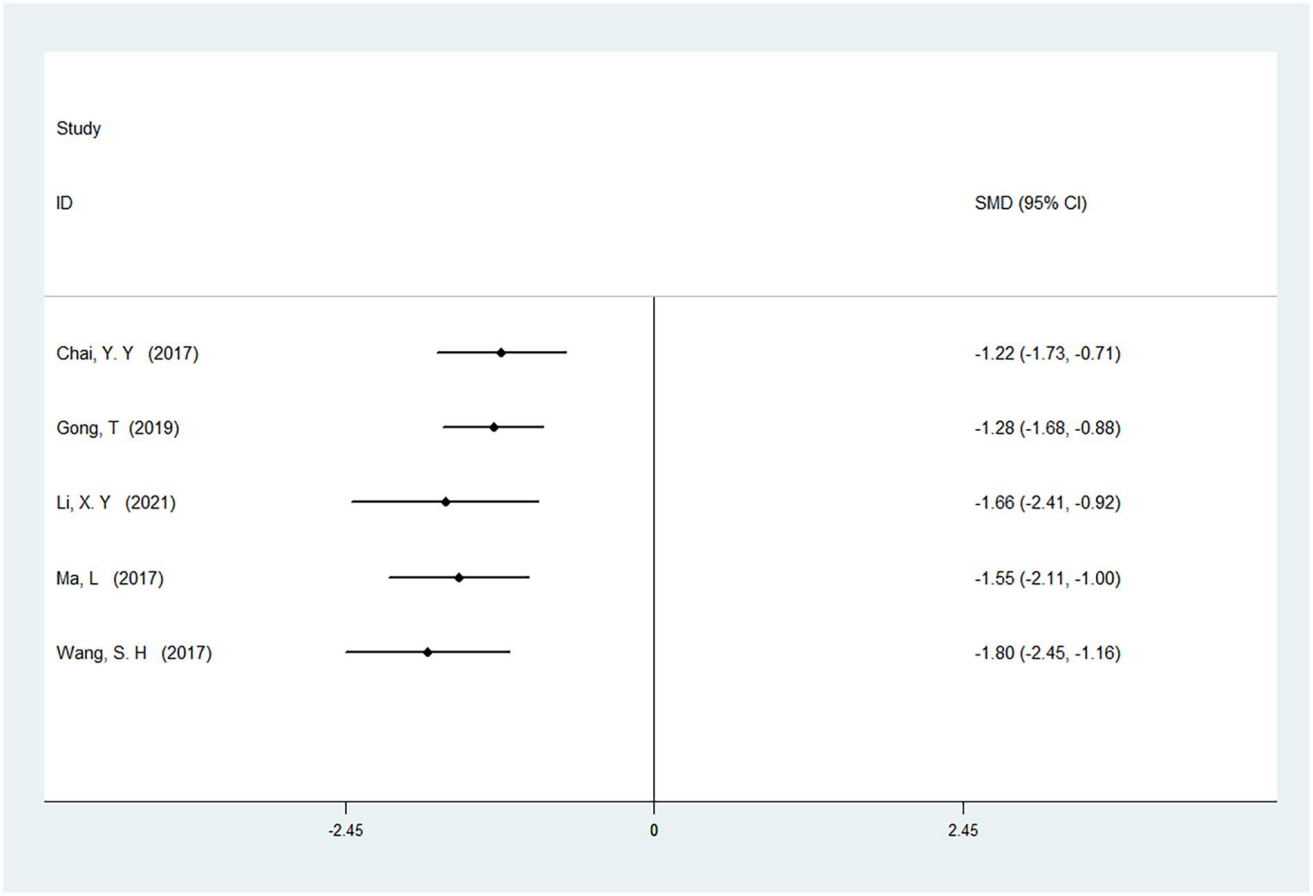
Cumulative meta-analysis forest map of time to first defecation.

A total of five studies were included to analyze the time to first defecation, involving 399 patients. The results showed high heterogeneity (*I*² = 86%, *P* < 0.01). There was a statistically significant difference in the time to first defecation between ERAS care and conventional care (SMD = −1.80, 95% CI = −2.45, −1.16; *P* < 0.05, see Table 4). Since the results for this outcome measure were all obtained from studies conducted in 2017, a cumulative meta-analysis was not performed, as detailed in Table 4. After cumulative analysis in chronological order of publication, the SMD value and the 95% CI tended to stabilize and showed a good trend of change. The first time when a statistically significant difference in shortening first defecation was confirmed was in 2015 [SMD = −1.80 (−2.45, −1.16)]. Subsequent studies narrowed the confidence interval, and the precision of the effect size gradually increased. This shows that ERAS care is superior to conventional care in shortening the first defecation. The GLS regression test showed that including all studies resulted in *P* = 0.00, and removing the first study resulted in *P* = 0.00. This suggests the presence of temporal variability in the results, indicating that the findings are not yet stable and necessitate further validation through additional clinical trials, as detailed in Table 6, Figure 11, and Supplementary Material S8.

#### 3.4.10 First-time out-of-bed activity

**Figure 12:**
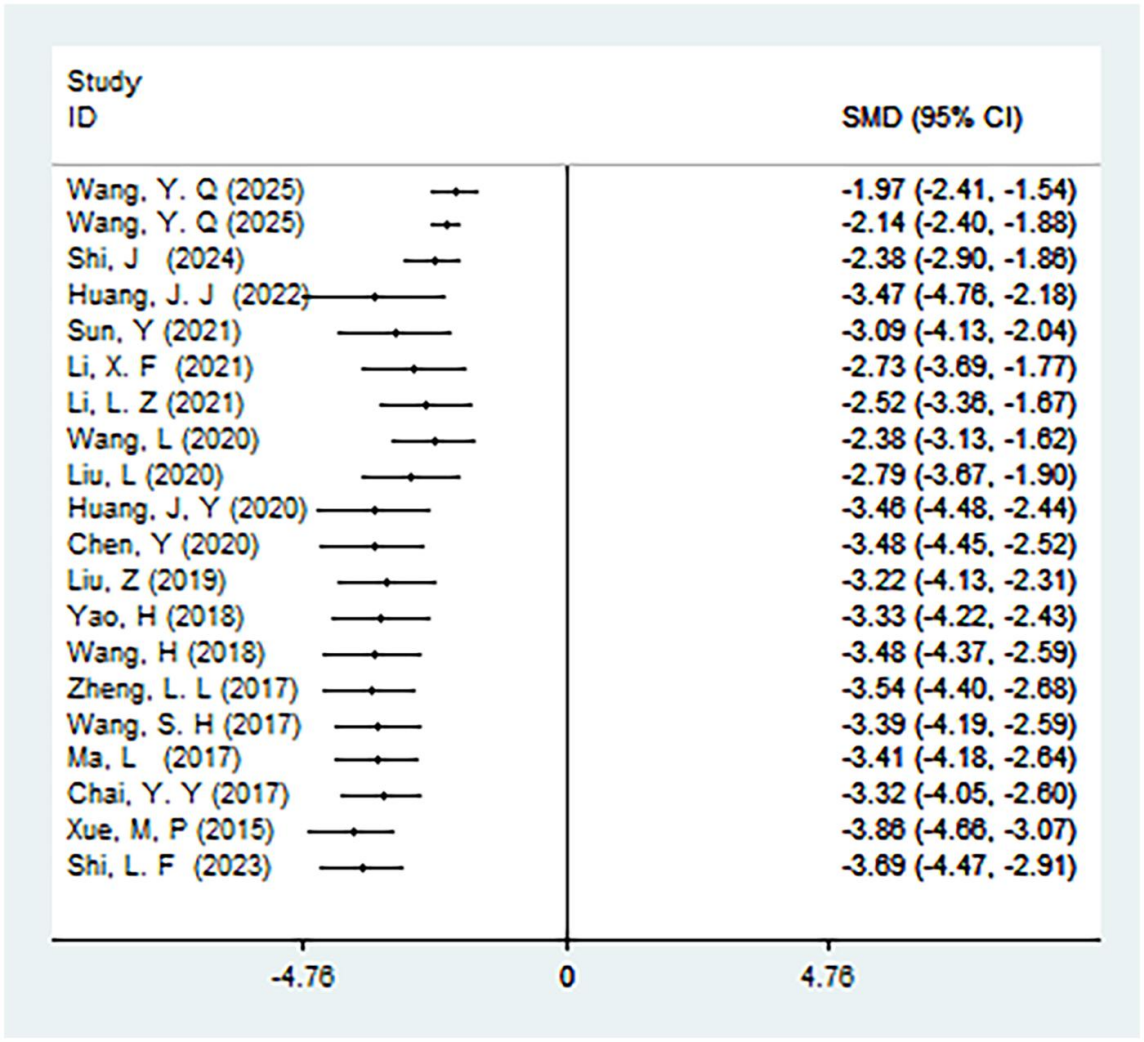
Cumulative meta-analysis forest map of the time to first time out of bed activity.

A total of 20 studies were included to analyze the first-time out-of-bed activity, involving 1,869 patients. The results showed high heterogeneity (*I*^2^ = 97%, *P* < 0.01). There was a statistically significant difference in the time to first-time out-of-bed activity between ERAS care and conventional care (SMD = −3.69, 95% CI = −4.47, −2.91; *P* < 0.05, see Table 4). Since the results for this outcome measure were all obtained from studies conducted in 2015, a cumulative meta-analysis was not performed, as detailed in Table 4. After cumulative analysis in chronological order of publication, the SMD value and the 95% CI tended to stabilize and showed a good trend of change. The first time when a statistically significant difference in shortening the first-time-out-of bed activity was confirmed was in 2015 [SMD = −3.69 (−4.47, −2.91)]. Subsequent studies narrowed the confidence interval, and the precision of the effect size gradually increased. This shows that ERAS care is superior to conventional care in shortening the first-time out-of-bed activity. The GLS regression test showed that including all studies resulted in *P* = 0.00, and removing the first study resulted in *P* = 0.00. This suggests the presence of temporal variability in the results, indicating that the findings are not yet stable and necessitate further validation through additional clinical trials, as detailed in Table 6, Figure 12, and Supplementary Material S9.

### 3.5 Sensitivity analyses

**Figure 13:**
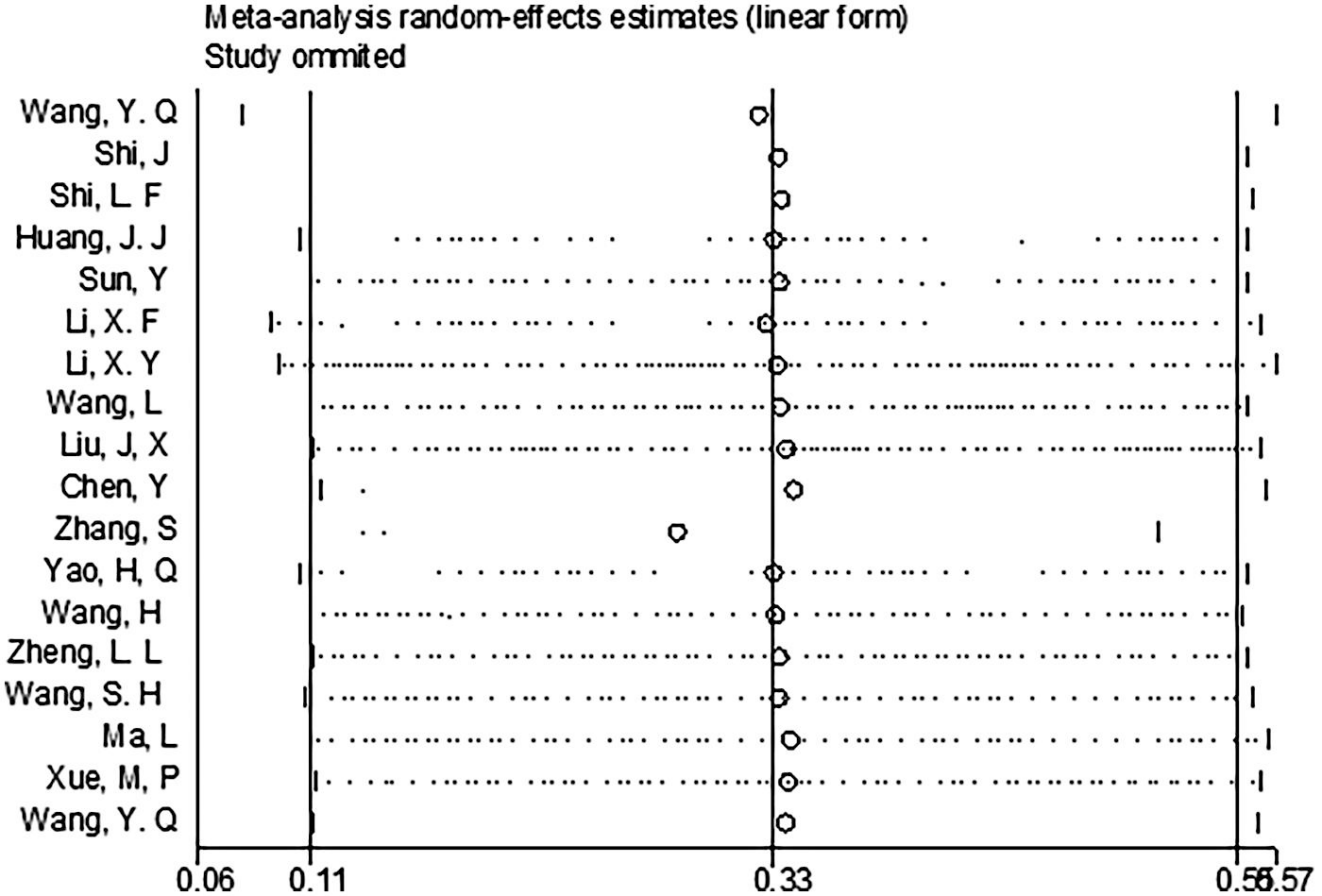
Sensitivity analysis of total complications.

Our study results show that after excluding most of the total complications, the time to first anal exhaust, time to first feeding, first urination time after surgery, removal time of drainage tube, length of hospital stay, catheter encumbrance time, satisfaction, time to first defecation, and first-time out-of-bed activity, the results of the remaining studies still have statistical significance, indicating that the original meta-analysis results are robust. A sensitivity analysis of the complications is illustrated in Figure 13, and the remaining results can be found in Supplementary Material S1–S9.

### 3.6 Publication bias

The Egger test and Begg's test for the time to total complications, first urination time after surgery, and time to first defecation found no publication bias (*P* > 0.05), while for the time to first anal exhaust, time to first feeding, removal time of drainage tube, length of hospital stay, catheter encumbrance time, satisfaction, and first-time out-of-bed activity, there was a possibility of publication bias (*P* < 0.05), as detailed in Table 7.

**Table 7:** Publication bias test.

Outcomes	Coefficient	Standard error	*t*	Egger's test (*P*)	Begg's test (*P*)
Total complications	−0.25	0.09	−2.66	0.19	0.23
Time of first anal exhaust	−7.82	2.65	−2.95	0.01	0.04
Time to first feeding	−14.03	2.51	−5.60	0.00	0.00
First urination time after surgery	−3.47	0.70	−4.93	0.13	0.30
Removal time of drainage tube	−12.11	2.04	−5.93	0.00	0.00
Length of hospital stay	−8.29	2.76	−3.01	0.01	0.01
Catheter encumbrance time	−12.68	2.36	−5.37	0.00	0.12
Satisfaction	2.84	0.45	6.34	0.00	0.00
Time to first defecation	−6.54	12.48	−0.52	0.64	0.22
First-time out-of-bed activity	−11.79	1.78	−6.61	0.00	0.00

### 3.7 GRADE certainty of evidence

GRADE evidence proﬁles for primary and secondary outcomes are given in Table 8. The certainty of evidence is moderate for total complications, low for first urination time after surgery and satisfaction, and very low for the time to first anal exhaust, time to first feeding, removal time of drainage tube, length of hospital stay, catheter encumbrance time, time to first defecation, and first-time out-of-bed activity.

**Table 8:** GRADE evidence proﬁles.

Outcomes	Risk of bias	Inconsistency	Indirectness	Imprecision	Publication bias	No of participants (studies)	Overall certainty of evidence
Total complications	−1^a^	0	0	0	0	1,865	Moderate
Time of first anal exhaust	−1^a^	−2^b^	0	0	−1^d^	2,232	Very low
Time to first feeding	−1^a^	−2^b^	0	0	−1^d^	1,181	Very low
First urination time after surgery	−1^a^	0	0	0	0	400	Low
Removal time of drainage tube	−1^a^	−2^b^	0	0	−1^d^	1,119	Very low
Length of hospital stay	−1^a^	−2^b^	0	0	−1^d^	2,071	Very low
Catheter encumbrance time	−1^a^	−2^b^	0	0	−1^d^	766	Very low
Satisfaction	−1^a^	0	0	0	0	1,008	Low
Time to first defecation	−1^a^	−2^b^	0	−1^c^	−1^d^	399	Very low
First-time out-of-bed activity	−1^a^	−2^b^	0	0	−1^d^	1,869	Very low

^a^ Random, hidden, or blind methods have drawbacks.

^b^ 50% < *I*^2^ < 75% after the combination of the included data.

^c^ Sample size was small (continuity variable <400, dichotomies <300).

^d^ There is publication bias.

## 4 Discussion

### 4.1 Summary of main findings

Our meta-analysis comprehensively and systematically reviews the currently available literature comparing the efficacy of ERAS care with conventional care in the recovery after laparoscopic radical nephrectomy. We found that compared with conventional care, ERAS care significantly reduced postoperative complications, shortened the time to first flatus, time to first feeding, time to spontaneous urination, time to drain removal, time to ureteral catheterization, and time to first bowel movement, and improved patient satisfaction. The analysis suggests that conventional preoperative bowel preparation not only causes discomfort to patients but may also lead to dysbiosis and electrolyte imbalance, thereby increasing intraoperative stress and affecting postoperative gastrointestinal recovery. The ERAS concept advocates for not performing bowel preparation preoperatively to alleviate patient suffering, reduce the risk of preoperative dehydration, thirst, hunger, and agitation, and avoid electrolyte disturbances and dysbiosis (48); early feeding can restore gastrointestinal function sooner, reduce fluid supplementation, help patients regain strength and nutrition, and maintain homeostasis, while early mobilization helps promote gastrointestinal motility, accelerate wound healing, and reduce the risk of deep vein thrombosis (49); early removal of various catheters can reduce discomfort caused by the catheters and decrease the risk of infections due to catheter retention (50). Conventional surgical trauma induces a significant neuroendocrine and inflammatory response, leading to a hypercatabolic state. The ERAS protocol alleviates surgical stress and systemic inflammation through multimodal interventions. After nephrectomy, the remnant kidney is exposed to compensatory hyperfiltration stress. ERAS contributes to the protection of residual renal function and reduces the risk of acute kidney injury via strategies such as goal-directed fluid management and early resumption of oral intake. Moreover, ERAS facilitates the recovery of gastrointestinal function and helps prevent postoperative ileus. The multimodal analgesia advocated by ERAS, which includes preoperative acetaminophen, intraoperative local anesthetic infiltration, and postoperative non-steroidal anti-inflammatory drugs combined with regional blocks, provides effective analgesia, while significantly decreasing opioid consumption and related adverse effects. On a technical level, ERAS integrates with laparoscopic minimally invasive techniques, refined pneumoperitoneum and drain management, and goal-directed perioperative fluid administration, thereby removing external barriers to recovery inherent in traditional care. Owing to these synergistic components, the ERAS pathway significantly reduces the overall incidence of complications and improves patient satisfaction following laparoscopic renal cancer surgery, ultimately promoting faster and safer recovery.

Our study results indicate the presence of time-related variability, and this instability does not occur randomly but exhibits a systematic pattern related to time (or study sequence). This suggests that there are essential differences between early and late studies. The analysis may indicate that early studies may have more methodological limitations (such as randomization, blinding, and analysis issues), while later studies adhere more closely to reporting standards like CONSORT, resulting in higher quality. Alternatively, there may be a temporal trend of clinical heterogeneity. The instability of the cumulative meta-analysis may reflect an overall improvement in methodological quality. The temporal variability of the cumulative meta-analysis is not the endpoint of the analysis; it is recommended that future systematic reviews/meta-analyses must not merely report an overall combined effect size. Time must be examined as a core variable. In conducting subgroup analyses and meta-regressions, “year of study publication” or “time period of study conduct” should be preset as an important covariate for analysis. Temporal variability may indicate that the conclusions of early studies are outdated. This suggests that new, larger original studies may be needed to verify the true effects in the current medical environment.

Our meta-analysis revealed substantial heterogeneity in several secondary outcome measures, which may be attributed to variations in the components of the ERAS protocols, specifically, differences in the specific items adopted and the intensity of implementation across the included studies. Furthermore, outcome measures such as the time to first flatus are subjective indicators that rely on patient-reported or nurse-elicited information, lacking objective and standardized measurement criteria. Such variability in assessment may also contribute to the observed high heterogeneity.

Our meta-analysis indicated potential publication bias for certain outcome measures, which may be attributed to the exclusion of a considerable number of lower-quality studies and the concentration of the included research on articles published by Chinese scholars.

### 4.2 Implication for clinical practice

The introduction of the ERAS concept greatly enriches the content of nursing, transforming the role of nurses from passive postoperative complication management to proactive rehabilitation facilitation. First, traditional views hold that nursing work mainly focuses on postoperative care, while ERAS advocates that the quality of preoperative nursing interventions directly determines the speed and extent of postoperative recovery. Therefore, nurses must strictly implement precise assessments and education, prerehabilitation guidance, and metabolic preparation. Second, traditional views consider postoperative pain to be normal, using analgesic pumps or morphine as needed. ERAS advocates that actively preventing pain is far more effective than passively treating it. Pain-free or minimally painful conditions are prerequisites for patients to mobilize early and cough effectively. Therefore, nurses should become coordinators of multimodal analgesia, finely assessing pain levels. Third, traditional views suggest that patients should rest well after surgery and wait to eat until they pass gas. ERAS advocates that early activity and feeding are “therapeutic measures” and not just recovery outcomes. They are key to preventing complications and promoting functional recovery. Therefore, nurses should develop targeted activity plans for patients, create conditions for activity, and actively encourage dietary recovery. Fourth, traditional views hold that drains and catheters should be retained for a long time as a precaution. ERAS advocates that every catheter is a potential infection pathway and a constraint on activity. Early removal of catheters is a key aspect of ERAS success. Therefore, after early removal of the urinary catheter, nurses should monitor the patient's ability to urinate spontaneously to prevent urinary retention. At the same time, encouraging early mobilization is itself the most effective nursing measure to prevent deep vein thrombosis in the lower limbs. Future research needs to conduct multicenter, large-scale validation in a broader geographical area and diverse medical system to further confirm the universality of the effect of ERAS care in laparoscopic renal cancer surgery.

### 4.3 Strengths and limitations

The strengths of this meta-analysis lie in its adherence to the PRISMA statement, registration of the protocol on PROSPERO, and the application of cumulative meta-analysis methods to assess the stability of the evidence at hand. Our meta-analysis has some limitations that may affect the interpretation of the results. First, the sample size of the studies included in the analysis is small, and some studies have unclear randomization methods and blinding. Second, although there is a high degree of heterogeneity in the secondary outcomes, it may be related to the differences in the included population and the ERAS protocol, and may affect the results. Third, there is insufficient reporting on postoperative analgesia and the cost-effectiveness or resource impact of implementing an electronic waste recycling system, which can be regarded as research directions in the future. Fourth, there is currently a lack of standardized ERAS protocols across studies, which urgently need to be improved by a nursing scholar. Finally, although we conducted a comprehensive search, the inclusion of studies was mainly concentrated in China, which might have limited the universality of the research results and their applicability to different medical systems and populations in other regions.

## 5 Conclusions

ERAS nursing is safe and effective for postoperative recovery following laparoscopic radical nephrectomy, promoting patient recovery. Given that the body of evidence is continuously evolving and there is time-related variability, systematic reviews on this topic should not be one-time efforts. A living systematic review mechanism should be established to regularly search for and incorporate new evidence, continuously update the cumulative meta-analysis to capture changes in conclusions in a timely manner, and provide the most up-to-date decision-making database for clinical practice.

## Data Availability

The raw data supporting the conclusions of this article will be made available by the authors, without undue reservation.
